# Bidirectional Functions of Arsenic as a Carcinogen and an Anti-Cancer Agent in Human Squamous Cell Carcinoma

**DOI:** 10.1371/journal.pone.0096945

**Published:** 2014-05-09

**Authors:** Nguyen Dinh Thang, Ichiro Yajima, Mayuko Y. Kumasaka, Masashi Kato

**Affiliations:** 1 Department of Biochemistry and Plant Physiology, VNU University of Science, Vietnam National University, Hanoi, Vietnam; 2 Department of Occupational and Environmental Health, Nagoya University Graduate School of Medicine, Nagoya, Aichi Prefecture, Japan; University of Kansas School of Medicine, United States of America

## Abstract

Bidirectional cancer-promoting and anti-cancer effects of arsenic for cancer cells have been revealed in previous studies. However, each of these effects (cancer-promoting or anti-cancer) was found in different cells at different treated-concentration of arsenic. In this study, we for the first time indicated that arsenic at concentration of 3 µM, equal to average concentration in drinking water in cancer-prone areas in Bangladesh, simultaneously expressed its bidirectional effects on human squamous cell carcinoma HSC5 cells with distinct pathways. Treatment with 3 µM of arsenic promoted cell invasion via upregulation of expression of MT1-MMP and downregulation of expression of p14ARF and simultaneously induced cell apoptosis through inhibition of expression of N-cadherin and increase of expression of p21(WAF1/CIP1) at both transcript and protein levels in HSC5 cells. We also showed that inhibition of MT1-MMP expression by NSC405020 resulted in decrease of arsenic-mediated invasion of HSC5 cells involving decrease in phosphorylated extracellular signal-regulated kinases (pERK). Taken together, our biological and biochemical findings suggested that arsenic expressed bidirectional effects as a carcinogen and an anti-cancer agent in human squamous cell carcinoma HSC5 cells with distinct pathways. Our results might play an important scientific evident for further studies to find out a better way in treatment of arsenic-induced cancers, especially in squamous cell carcinoma.

## Introduction

Arsenic contamination in drinking well water is a serious public health problem in the world [1], [2]. Arsenic is a well-documented human carcinogen. Chronic low-dose exposure of arsenic caused skin discoloration, chronic indigestion, hypertension, peripheral vascular disease, ischemic heart disease, and many types of cancers including skin, lung, bladder, liver, and kidney cancers [3]. Effects of arsenic as an agent for carcinogenesis or tumor progression or an anti-tumor drug depend on its concentration [4]–[6], duration of exposure [6], [7] and cancer cell types [3]–[7]. Previous auto radiographic animal studies showed that cutaneous squamous cell carcinoma (SCC) is one of the representative arsenic-mediated cancers [8]. Although arsenic has been widely recognized as a carcinogen, it also has been clinically used as an effective chemotherapeutic agent in treatment of leukemia in humans [9]. Arsenic also expressed its anti-cancer effects on various solid cancers, including cutaneous carcinoma, through promoting apoptotic cell death [10], [11]. The bidirectional cancer-promoting and anti-cancer effects of arsenic on cancer cells have led to a difficult situation to clarify the mechanism of arsenic-mediated cancer. Although there were many studies focusing on revealing the mechanism of effects of arsenic on cancer cells, it is still unclear.

Arrest of the cell cycle and apoptosis are related to cell death and to be considered as important ways for cancer treatment [12]–[15]. Previous studies showed that arsenic induces apoptosis in cancer cells via activation of expression of tumor suppressors of p21(WAF1/CIP1) and p14ARF (p19ARF in mouse) [12]–[15]. p21(WAF1/CIP1) and p14ARF play important roles in controlling the cell cycle arrest by regulating the activity of cyclins and cyclin-dependent kinases (CDK) [16]–[19]. p21(WAF1/CIP1) and p14ARF are able to inhibit cell growth through cell cycle arrest of skin cancer cells including melanoma, squamous cell carcinoma and basal cell carcinoma [20]–[24]. Other reports revealed that exposure to arsenic cause cell transformation through the inhibition of both protein and gene expression of the tumor suppressors p19ARF [22]–[24].

Invasion is hall mark for malignancy grade of cancer cells. It is reported that arsenic reduces the invasive and metastatic properties of glioma tumor cells via inhibition of activation of matrix metalloproteinase-14 (MT1-MMP) [7], [25], which is able to drive invasion of cancer cells largely by degrading ECM barriers. MT1-MMP is also considered as a upstream of ERK. ERK is a key molecule in the major signaling cassettes of the mitogen-activated protein kinase pathway [26], . ERK plays an important role in cancer development, [26], [27]. Thus MT1-MMP may be able to regulate the phosphorylated level of ERK [26], [27]. However, in the other studies, high concentration of arsenic enhances MT1-MMP expression in fibroblast cells [28]–[30]. Previous studies also reported that arsenic reduces expression of E-cadherins [31], [32]. Downregulation of E-cadherin is correlated with upregulation of N-cadherin, an invasion promoter molecule [33]–. N-cadherin–dependent adhesion impairs the upregulation of the cyclin-dependent kinase inhibitor p21 [37], [38]. Ectopic expression of N-cadherin increases tumor cell motility [35], [39].

Our previous report showed that there is about 3 µM (210.7 µg/L) of arsenic in the arsenic-polluted drinking well water (n = 72) in cancer-prone areas in Bangladesh [40]. There are many types of cancers including squamous cell carcinomas (SCC) occurring in these areas [40]. In this study, we for the first time showed that arsenic at concentration of 3 µM simultaneously acted as cancer-promoter and anti-cancer drug in human squamous cell carcinoma HSC5 cells with distinct pathways.

## Materials and Methods

### Reagents

Sodium arsenide (arsenic) was purchased from Sigma. NSC405020 was purchased from Millipore. Arsenic was dissolved in water for use. NSC405020 was dissolved in DMSO for use.

### Cell culture

Human transformed keratinocytes HSC5 cells [40] (Health Science Research Resources Bank, Japan) were cultured in RPMI-1640, supplemented with 10% Fetal Bovine Serum (FBS) and 1% Penicillin/Streptomycin at 37°C in 5% CO2.

### Crystal violet assay

Crystal violet assay was performed using the method previously described [40]. Briefly, cells (3×10^4^ cells) were plated in six-well plates and cultured for 24 h. Cells were then treated with arsenic and cultured for a further 3 days. The viable adherent cells were fixed with 10% formalin and stained with 0.1% crystal violet. Absorbance at 595 nm in the stained cells solubilized with 0.1% SDS was measured using a microplate reader.

### Invasion assay

Cell invasion ability was evaluated by invasion assay according to the method previously reported [41]. Briefly, 2×10^5^ cells in 300 ml culture medium with 0.5% FBS were applied to the matrigel-coated upper chamber of 8 mm in diameter (8 mm in pore size). Then the upper chambers were placed in 24-well culture plates containing 600 ml conditioned medium with 0.5% FBS to trigger invasion activity and were incubated for 12 hours. Invading cells were stained with hematoxyline-eosine or crystal violet and counted under a microscope.

### Real-time PCR analysis

Total RNA was prepared from cell line samples using a High Pure RNA Kit (Roche Diagnostics) according to the method previously described [41]. cDNA was then synthesized by reverse transcription of total RNA using Super-criptTMIII reverse transcriptase included in the RT enzyme mix and RT reaction mix according to the protocol previously described [41]. Real-time quantitative RT-PCR with SYBR green was performed using power SYBR1 Green PCR master mix (Applied Biosystems) in an ABI Prism7500 sequence detection system (Applied Biosystems). The expression levels of p14ARF, p21(WAF1/CIP1), MT1-MMP and N-cadherin transcripts measured by quantitative RT-PCR (real-time PCR) were adjusted through the transcript expression level of TATA-box-binding protein (TBP). PCR was carried out using 10 ml of power SYBR1 Green PCR master mix (Applied Biosystems) containing 900 nM forward primer and 900 nM reverse primer in a final volume of 20 ml. Sequences of primers are presented as below:

Forward: GTGGTCTCGGACCATGTC

and Reverse: GTAGCCATATTGCTGTAGCC for MT1-MMP.

Forward: ATTGCTGTTTTGGACCGAGA

and Reverse: CACTTGAGGGGCATTGTCAT for N-cadherin.

Forward: AAGTCAGTTCCTTGTGGAGC

and Reverse: ATTAGCGCATCACAGTCGCG for p21(WAF1/CIP1).

Forward: ATGGTGCGCAGGTTCTTGGT

and Reverse: TGCCCATCATCATGACCTGG for p14ARF.

Forward: CACGAACCACGGCACTGATT

and Reverse: TTTTCTTGCTGCCAGTCTGGAC for TBP.

### Immunoblot analysis

Immunoblot analysis was performed according to the method described previously [42]. Rabbit polyclonal first antibodies against phosphorylated threonine 202 in ERK1 and phosphorylated tyrosine 204 in ERK2 (Cell Signaling), anti-matrix metalloproteinase-14 (MT1-MMP) hinge region antibody (Millipore), ERK1/2 (Cell Signaling), Goat polyclonal antibodies against p14ARF and p21(WAF1/CIP1) (Santa Cruz), mouse monoclonal antibody against N-cadherin (*BD* Biosciences ) and mouse monoclonal antibody against alpha-TUBULIN (SIGMA).

### Statistical analysis

Statistical analysis in this study was performed according to the method previously described [40]. Results from three independent experiments in each group were statistically analyzed by Student's t-test. The SPSS (version 18) software package (SPSS Japan Inc.) was used for these statistical analyses, and the significance level was set at p<0.05.

## Results

### Arsenic induced apoptosis of HSC5 cells

**Figure 1:**
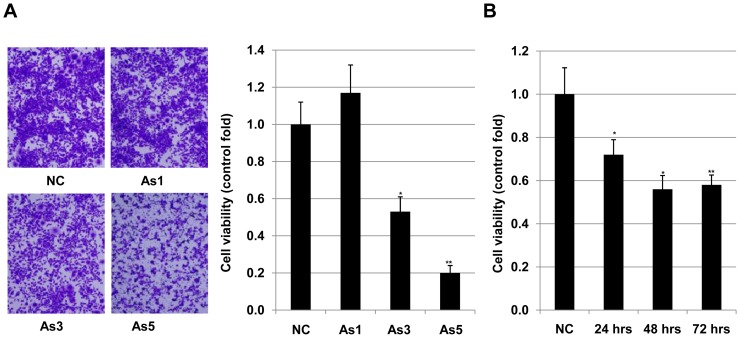
Effects of arsenic on apoptosis of HSC5 cells. Viability of HSC5 cells treated with 0, 1.0, 3.0 and 5.0 µM of arsenic was evaluated by crystal violet (CV). Cells were presented in photographs (A) and ratios of arsenic treated live cells and control live cells were presented in a graph (B). **, Significantly different (p<0.01) from the control by the Student's t-test.

Our previous fieldwork showed that concentration of arsenic in arsenic-polluted drinking water in cancer-prone areas in Bangladesh is about 3 µM (210.7 µg/L) [40]. However, there is a fact that it is difficult to identify the effect of arsenic on cancer development [3]–[11] therefore we decided to investigate of effects of arsenic on apoptosis of HSC5 cells. HSC5 cells were treated with arsenic at 0, 1.0, 3.0, 5.0 and 10.0 µM for 24 hrs. It showed that the higher concentration of arsenic caused the stronger apoptosis of the cells. At concentration of 1 µM, arsenic had no effect on cell dead (Figure 1A). However, at 3 µM and 5 µM, arsenic caused decrease of cell viability almost 2 and 5 folds, respectively (Figure 1A). 10 µM of arsenic led to almost all cells dead (data not shown). We then investigated the effect of 3 µM of arsenic on the dead of HSC5 cells with a time course. Treatment with arsenic for 24 hours, 48 hours and 72 hours significantly decreased viability of HSC5 cells 1.4, 1.8 and 1.7 times, respectively (Figure 1B). These results indicated that apoptosis of HSC5 cells were directly caused by arsenic.

### Arsenic promoted invasion of HSC5 cells

**Figure 2:**
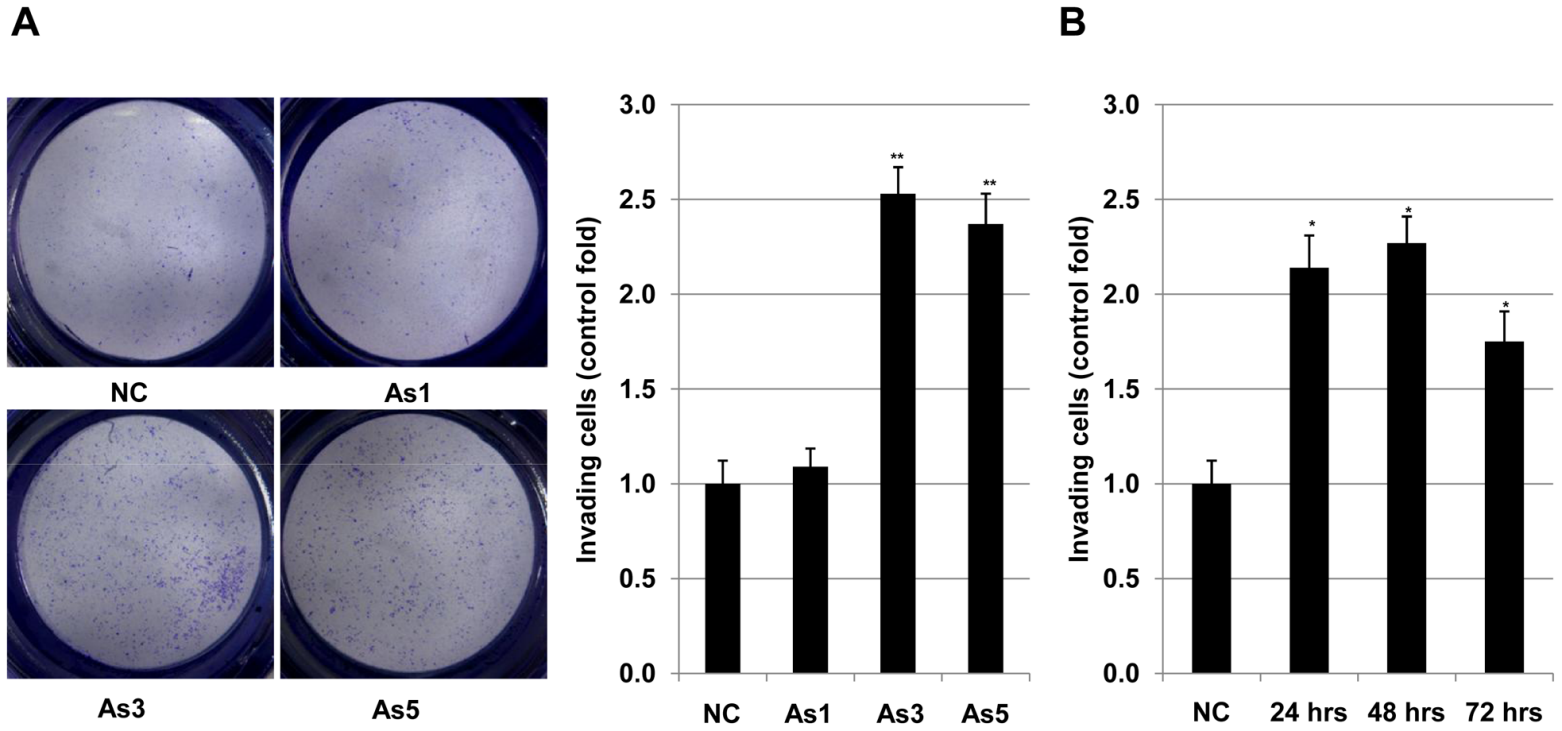
Effects of arsenic on invasion of HSC5 cells. Invasive ability of of HSC5 cells treated 0, 1.0, 3.0 and 5.0 µM of arsenic were evaluated by invasion assay. Number of invading HSC5 cells treated with arsenic in the invasion assay were presented in photographs (A) and a graph (B). **, Significantly different (p<0.01) from the control by the Student's t-test.

We next examined effects of arsenic on invasion of HSC5 cells. Cells were pre-treated with arsenic at different concentrations (0, 1.0, 3.0, 5.0 and 10.0 µM) for 24 hours before harvesting for invasion assay. Treatment with 1 µM of arsenic had no effect, however 3 µM and 5 µM promoted invasion of HSC5 about 1.8 and 3.7 folds, respectively (Figure 2A). At 10 µM, arsenic caused the dead of almost all cells, therefore it was not possible to investigate the effect of arsenic on invasion of cell at this concentration. We then investigated the effect of 3 µM of arsenic on invasion of HSC5 cells with a time course of 0, 24, 48 and 72 hours. Pre-treated with arsenic for 24, 48 and 72 hours increased invasion of HSC5 cells 2.14, 2.27 and 1.75 folds, respectively (Figure 2B). There results suggested that arsenic resulted in promotion of invasion of HSC5 cells. Treatment with 3 µM or 5 µM of arsenic increased apoptosis (Figure 1) and simultaneously promoted invasion of HSC5 cells (Figure 2). These results are scientific evidents for bifunctions of arsenic as a carcinogen and anti-cancer agent in cancer squamous cell carcinoma HSC5 cells. Basing on these results and the data from the fieldwork, we decided to use 3 µM of arsenic for further experiments.

### Arsenic promoted invasion of HSC5 cells by upregulation of MT1-MMP and downregulation of p14RAF at both transcript and expression levels

**Figure 3:**
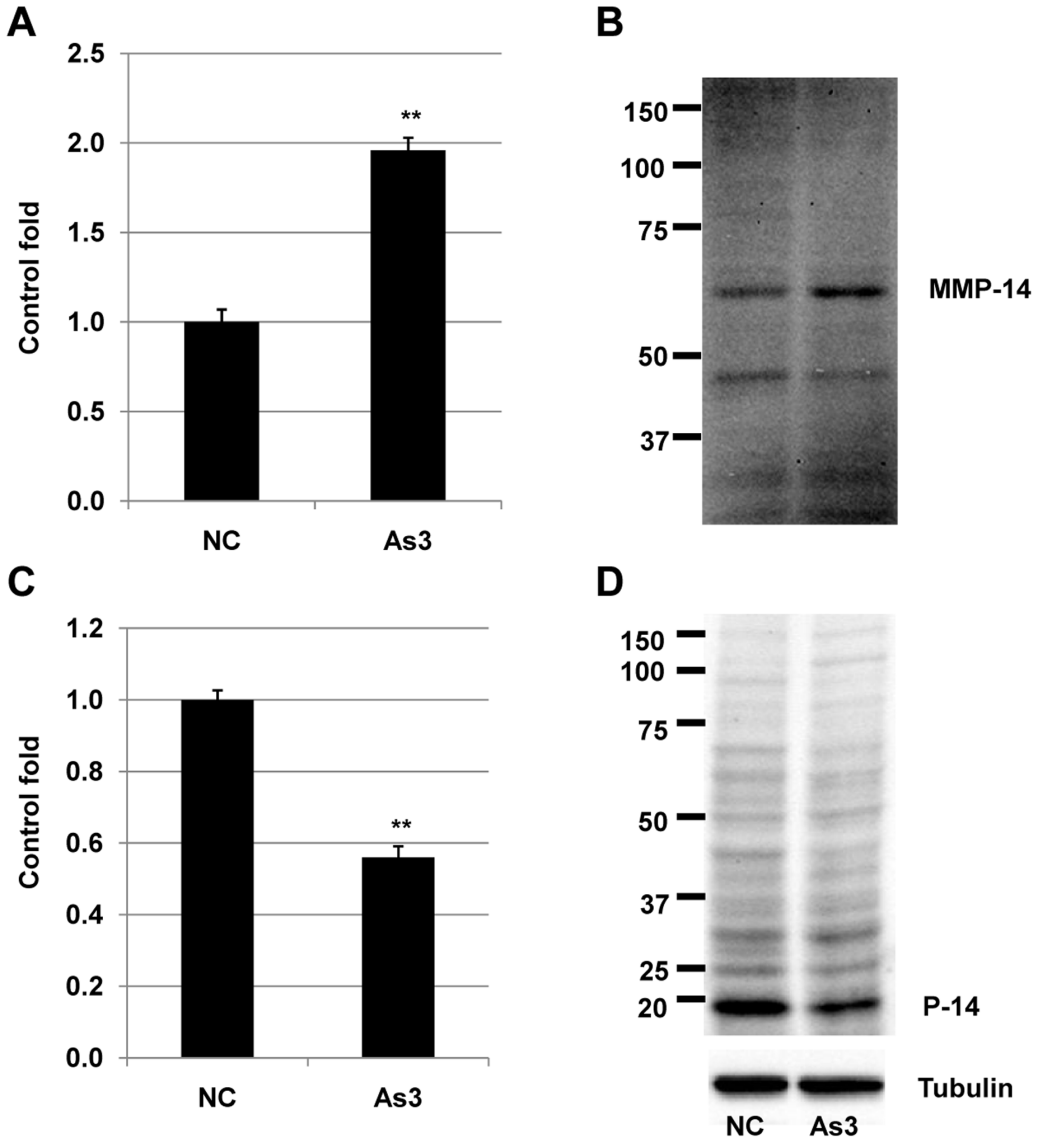
MT1-MMP and P14 transcript and protein expression levels in HSC5 cells treated with arsenic. A and C) transcript expression levels of MT1-MMP and p14 were measured by real-time PCR. **, significantly different (p<0.01) from the control by Student's t-test. B and D) Protein expression levels of MT1-MMP and p14 were measured by immunoblot. TUBULIN was used as a positive control. Three independent experiments were performed and the same results were obtained.

We next examined the molecular mechanism of arsenic-mediated cellular invasion in HSC5 cells. Treatment with 3 µM of arsenic induced transcript and expression levels of MT1-MMP (Figure 3A-B) and inhibited transcript and expression levels of p14ARF (Figure 3C-D). Previous studies [20]–[24], [27] indicated that MT1-MMP and p14ARF might play important roles in modulating growth and invasion of squamous cell carcinoma cells. In accordance with these previous reports [20]–[24], [27], our results suggested that arsenic might promote invasion of HSC5 cells via upregulation of MT1-MMP and downregulation of p14ARF.

### Arsenic induced apoptosis of HSC5 cells by downregulation of N-cadherin and and upregulation of p21(WAF1/CIP1) at both transcript and expression levels

**Figure 4:**
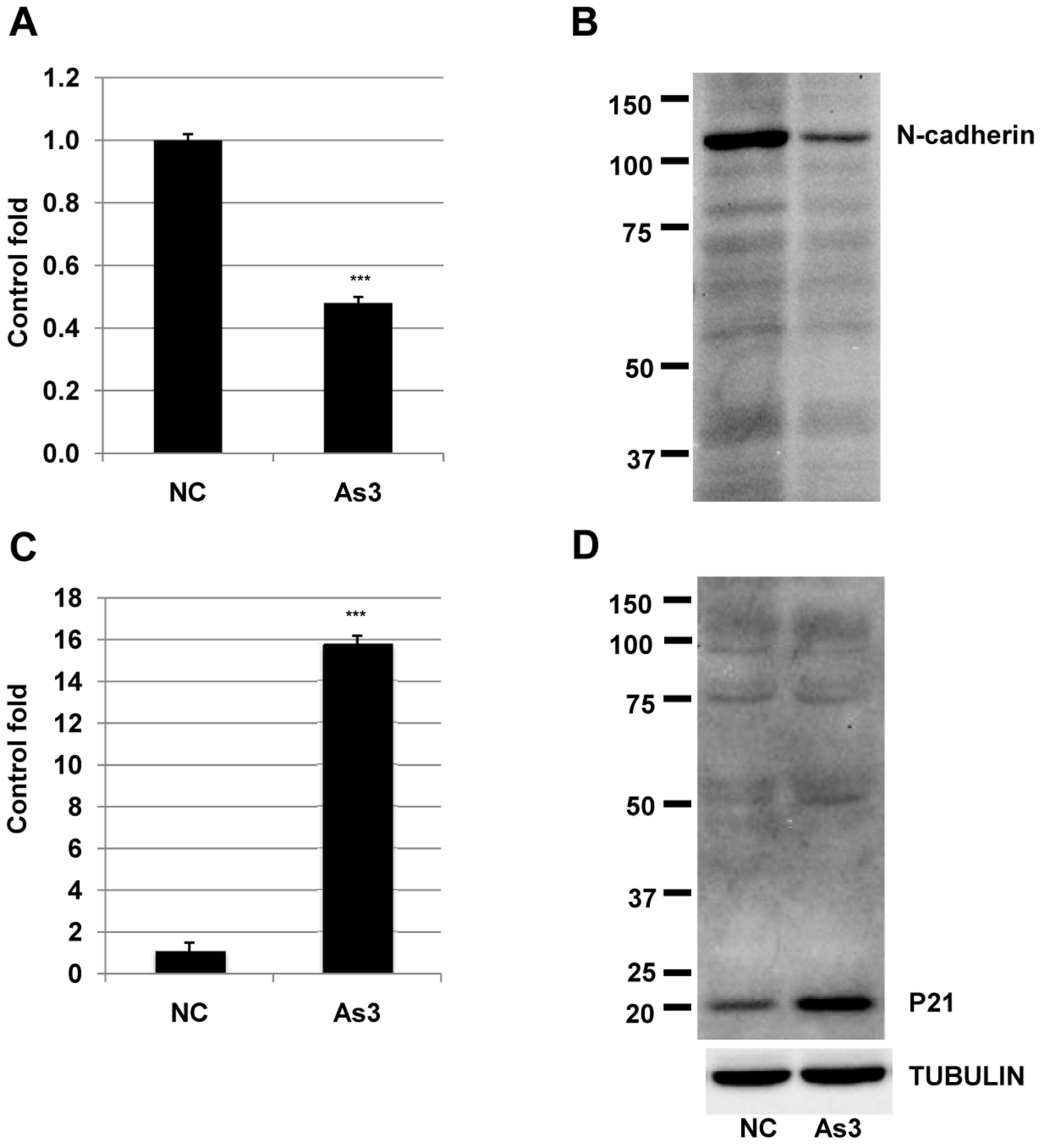
N-cadherin and p21 transcript and protein expression levels in HSC5 cells treated with arsenic. A and C) transcript expression levels of N-cadherin and p21 were measured by real-time PCR. ***, significantly different (p<0.001) from the control by Student's t-test. B and D) Protein expression levels of N-cadherin and p21 were measured by immunoblot. TUBULIN was used as a positive control. Three independent experiments were performed and the same results were obtained.

We then investigated the molecular mechanism of arsenic-mediated apoptosis in HSC5 cells. Treatment with 3 µM of arsenic strongly reduced transcript and expression levels of N-cadherin (Figure 4A-B) and promoted transcript and expression levels of p21(WAF1/CIP1) (Figure 4C-D). Previous studies [20]–[24], [29]–[36] showed that N-cadherin and p21(WAF1/CIP1) might play important roles in modulating of apoptosis of human squamous cell carcinoma cells. Our results in this study suggested that arsenic might cause apoptosis of HSC5 cells through downregulation of N-cadherin and/or upregulation of p21(WAF1/CIP1).

### Inhibition of arsenic-mediated promotion of invasion HSC5 cells by a MT1-MMP inhibitor

**Figure 5:**
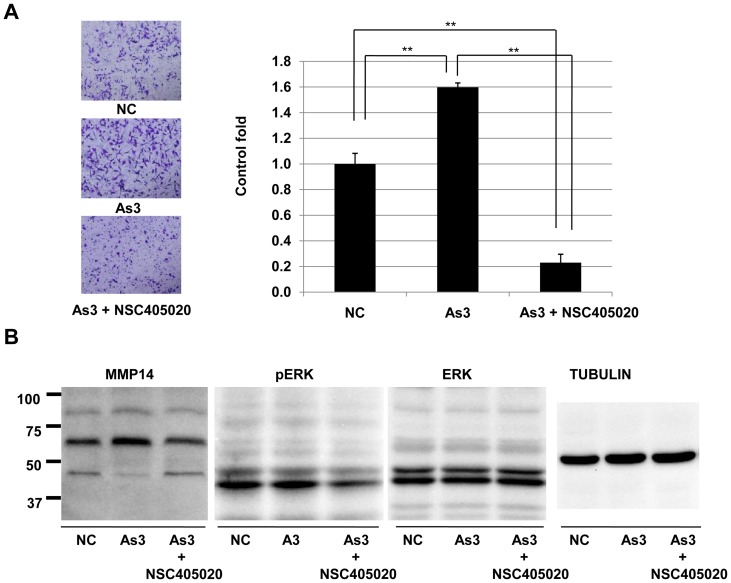
Effects of arsenic on invasive activity and phosphorylation and/or expression levels of MT1-MMP and ERK 4 in HSC5 cells. A), Invasive activity of HSC5 treated with 3 µM of arsenic was evaluated invasion assay. Level of invasive ability is presented as number of invading cells in a graph (left) and photographs (right). **, Significantly different (p<0.01) from the control by the Student's t-test. Phosphorylated levels of ERK (P-ERK) and protein expression levels of MT1-MMP and ERK in HSC5 cells treated with 3 µM arsenic for 24 hours are presented. TUBULIN protein expression levels are presented as an internal control.

We next examined the effect of NSC405020, a MT1-MMP inhibitor, on arsenic-mediated invasion of HSC5 cells (Figure 5). Since MT1-MMP has been reported to be potential sited upstream of ERK [41], [42] and may be associated with arsenic-mediated invasion (Figure 2). Treatment with 3 µM arsenic again increased invasion (Figure 5A) with an increase in expression level of MT1-MMP (Figure 5B). However, there was no change in the phosphorylated level of ERK (pERK). These results indicated that the bidirectional effects of arsenic at this concentration on pERK were balance. Arsenic-mediated invasion was blocked by treatment with 1 µM of NSC405020 (Figure 5A). NSC405020 (1 µM) caused to decrease of expression level of MT1-MMP as well as decrease of phosphorylated of ERK in HSC5 cells (Figure 5B).

## Discussion

Arsenic has been considered as an agent for carcinogenesis and tumor progression for a long time ago [1], [2]. Based on the analysis of 52,202 hand tube-well water samples during the last 14 years in Bangladesh showed that around 36 million and 22 million people could be drinking As-contaminated water above 10 and 50 µg/l, respectively [43]. This may be the main cause for serious problem of cancer development, especially for skin cancer in Bangladesh [3]. In a contrast way, arsenic also has been used as a drug for treatment of many types of cancers [8]–[11]. Basing on our previous result [40], we examined the effects of 3 µM arsenic on cellular invasion, a hallmark of malignancy grade of cancer cell and apoptosis, a marker for cell dead in cancer treatment. Our result showed that arsenic simultaneously strongly induced apoptosis (Figure 1) and promoted invasion (Figure 2) of HSC5 cells. These results suggested that at this concentration, arsenic expressed its bidirectional functions in squamous cell carcinoma HSC5 cells. We then investigated the molecular mechanisms related to these events in HSC5 cells. Our results revealed that 3 µM arsenic enhanced cellular invasion through upregulation of membrane type 1 matrix metalloproteinase (MT1-MMP) (Figure 3A), which plays crucial roles in tumorigenesis [7], and downregulation of p14ARF (Figure 3B), which is an inhibitor for cell proliferation [20]–[24] at both transcript and expression levels. This is the first time we showed that there is a possibility in cooperation between MT1-MMP and p14ARF in cancer development in HSC5 cells. In the other hand, our results showed that 3 µM arsenic induced apoptosis of HSC5 cells via downregulation of N-cadherin (Figure 4A), which plays role in cell differentiation, transformation, as well as invasion [33]–[36] and upregulation p21(WAF1/CIP1) (Figure 4B), a tumor suppressor [20]–[24] at both transcript and expression levels. Our results in accordance with previous studies [37], [38] suggested that N-cadherin might participate to the associated-cell cycle arrest through the nuclear accumulation of cyclin-dependent kinase inhibitors p21. Although both p14ARF and p21(WAF1/CIP1) were reported as molecules which play important roles in controlling the cell cycle arrest by regulating the activities of cyclins and cyclin-dependent kinases (CDK) [16]–[19], in this study, these molecules expressed their distinct effects on invasion and apoptosis of HSC5 cells. Further, we showed that NSC405020, a MT1-MMP inhibitor, inhibited the arsenic-mediated promotion of invasion HSC5 cells (Figure 5). It can be explained that treatment with NSC405020 resulted in decreasing of expression level of MT1-MMP. In turn, MT1-MMP regulated the phosphorylated level of extracellular signal-regulated kinases (pERK) [26], [27], which plays an important role in cellular invasion, proliferation and tumor development [26], [27]. Finally, downregulation of MT1-MMP and pERK by NSC405020 led to decreasing the invasion of HSC5 cells (Figure 5). Bidirectional cancer-promoting and anti-cancer effects of arsenic for cancer cells cells have been revealed. However, arsenic as a cancer-promoting agent or an anti-cancer drug was found in different cells at different treated-concentration in a particular report [1]–[3], [8]–[11]. This is the first time we simultaneously showed the bidirectional functions of arsenic in human squamous cell carcinoma HSC5 cells with distinct molecular pathways. This study helped to explain the reason why although there are many people have been exposed to arsenic, not all of them have arsenicosis diseases including cancers. Our results provided an important information for other studies in the future to find out a better way in treatment of arsenic-induced cancers, especially in squamous cell carcinoma.
